# The periodicity and initial evolution of micro-mobility systems: a case study of the docked bike-sharing system in New York City, USA

**DOI:** 10.1186/s12544-022-00549-y

**Published:** 2022-06-21

**Authors:** Liye Zhang, Jie Song

**Affiliations:** 1 College of Transportation, Shandong University of Science and Technology, Qingdao, 266590 Shandong China; 2 Institute of High Performance Computing, Agency for Science, Technology, and Research, 138632 Singapore, Singapore

**Keywords:** Docked Bike-sharing system, Principal component analysis, Spatiotemporal

## Abstract

**Objectives:**

This study developed an analytical framework that aims at understanding the evolutionary processes of a micro-mobility system (for example, bike-sharing), which offers insights into the transforming nature of a city transport system.

**Methods:**

Firstly, the framework applied a Gaussian Mixture Model to examine the long-term fluctuations of travel demands. Secondly, it investigated the growth trajectories of service points via exponential and logistic growth models. Cumulative connections with other points represented the growth of a service location. An eigendecomposition approach was used to uncover the hidden structures behind the growth curves.

**Results:**

This framework was applied in the docked bike-sharing program in New York City, USA. The results show that there existed periodic patterns of travel demands in the long term. The majority of stations grew rapidly after they began to operate. However, the temporal signatures of stations’ growth displayed some variations across different locations.

**Conclusion:**

This proposed workflow can be employed in other cities with similar context to better investigate how micro-mobility systems evolve.

## Introduction

With the introduction of micro-mobility services, the landscape of modern urban transport is evolving. These new services have provided flexible commuting modes to fulfill human mobility needs. There are a wide range of micro-mobility options, including shared bikes, e-scooters, and e-bikes. Bike-sharing systems, as an early micro-mobility alternative, have played an essential role in urban transportation ecosystems. They help to mitigate traffic congestion [19], lower fuel consumption [65] and carbon footprint [63], and benefit human health [49]. Bike-sharing systems also evolve rapidly. And newer generations of systems are being developed and improved constantly [36]. Early systems are station-based, and the latest generation is fully dockless. Both docked and dockless programs have been spreading across the world. Until August 2020, bike-sharing services are in operation in 2005 cities with more than 9 million public bikes, and 385 cities are currently deploying a bike-sharing system [40]. Recent years have also witnessed a boom of e-bike sharing systems. E-bikes are more convenient and less laborious than traditional bikes, thus many people choose e-bikes as a replacement of some conventional modes such as bus and private driving. However, e-bike riders can be exposed to greater risks than cyclists because these power assisted devices have higher speed variations and vibration impact than bikes [37].

Micro-mobility systems pose both challenges and opportunities for the management of modern cities. A docked bike-sharing system, for example, is different from public transit that has fixed routes and schedules. Moreover, it is also unlike personal or private-hire vehicles regarding routing schemes, costs, and usage patterns. Thus, most previous studies have mainly focused on understanding its operation and user behavior. There is a proliferation of relevant publications regarding the fluctuations of travel demands [11, 25], spatiotemporal usage patterns of cycling activities [28, 51], and the optimization of bike-sharing schemes [12]. Another line of inquiries is to study the dynamics of micro-mobility systems. Because they are an integral part of urban systems, their interactions with built environments and other transport modes are of great interests [34, 42]. More importantly, when there is a public health crisis (such as COVID-19 outbreak), shared bikes are preferred by many commuters who normally take bus or train [56]. Unfortunately, traditional urban planning and transportation modeling practices insufficiently take into account micro-mobility system and its growth, which in turn can also be an opportunity to enrich modern transportation and planning theories and practices.

Romanillos et al. [44] points out several priorities for future research of system sciences, one of which is to evaluate the growth of a system and its impacts. The introduction of micro-mobility sharing will likely shape urban landscape across the globe in the future, but its long-term growth patterns are still under-researched. Hence, this study aims at developing a methodological framework to recognize the network evolution of a micro-mobility system. The framework intends to detect overall periodic patterns of the system via a Gaussian Mixture Model clustering approach, and employs exponential and logistic models to explore the growth trajectories of bike stations. An eigendecomposition approach is employed to unravel the hidden structures of the trajectories. The proposed framework was applied using collected cycling trips from Citi Bike, a docked bike-sharing scheme in New York City, USA. The main contributions of this work lie in the development of an integrated framework and the reconstruction of the growth trajectories of a micro-mobility system through a simple process based on the eigendecomposition method.

The rest of this paper is organized with the following sections. Section 2 provides a general review on the topics of the evolution of social, transportation, and micro-mobility networks. Section 3 describes the framework and its underlying methodologies. Section 4 presents case study results. Subsequently, Sect. 5 extends our discussions on emerging findings and concludes this study with potential limitations and future research directions.

## Related works

Investigating the evolution of transportation networks (including micro-mobility systems) is a worldwide academic interest (Tables 1 and 2). This section reviews past efforts regarding the growth of transportation and micro-mobility networks in two relevant aspects. First, we summarize the concepts of network growth and its applications in the social and transportation sciences because these are early endeavors on probing evolving networks. Second, we present recent studies on the evolution of micro-mobility systems. Specifically, related works are organized into tables to highlight their geographical context and relevance to this study (Tables 1 and 2).

### Modeling the growth of social and transportation networks

Network evolution began to draw scientific attention when people observed a power-law distribution in web, citation, and social interaction networks. The preferential attachment model and its extensions have been developed to reproduce the power-law phenomenon by assuming that new links are more likely to attach to the nodes with high degrees [1]. These models have surprisingly high precision and a concise mathematical formation. Therefore, preferential attachment-based approaches have been applied in empirical studies about social networks. For example, Kossinets and Watts [29] investigated an evolving social network of over 40,000 students and faculty in a university and modeled how they interacted via emails. They found that, on average, the network properties were stale over time. Similarly, Kumar et al. [30] applied a modified preferential attachment model into an online social network consisting of 5 million individuals. The authors identified a stable network structure with three major components: singletons, communities, and giant components.

Physicists have also devoted efforts to understanding the topological properties of evolving transportation networks (Table 1). Transportation networks are different from other small-world networks because the former is situated in a constraint geographical location [14, 20]. However, a transportation network may share common properties with non-spatial counterparts if viewed from the perspective of network growth [31]. The modeling of evolving transportation networks has become a subject of intense academic scrutiny for more than seven decades. Previous efforts are within three main streams: the geography of network growth, the correlations among growth properties, traffic flows, and transport policies, and the analyses of topological properties [59]. Surface transportation networks such as roads and highways can be naturally thought of as evolving networks. First, early attempts have centered at simulating continual transformations of road networks that have geographical constraints. Taaffe et al. [52] first described a four-step model for road development in a series of discretized stages. This fourstage framework assumed that the connections and penetrations between seaports and inland trading points facilitated road links. This classic model was later adopted in other case studies under different spatial settings [41, 43]. Transportation networks are parts of an urban system, and they are inter-connected with the other sub-components of a city. Secondly, network growth is driven by the rational decisions of travel users, infrastructure suppliers, developers, and policy initiators. Traffic flows are believed as a critical role in shaping network geometries [16]. Many interesting phenomena of evolving transportation networks have been discovered as well. For instance, Cervero and Hansen [10] observed the 20-year changes of road networks in California, USA, and found that there existed reciprocal relationships between vehicle miles traveled and road supply. Likewise, Levinson [33] and Taylor et al. [53] discovered similar mutual effects between transit demand and supply. In conclusion, phenomenal interests have been attracted to the domain of growing transportation networks. There are numerous successful applications of the concepts of preferential attachment models that emerged earlier in natural sciences.

**Table 1:** Global studies investigating the growth of transportation networks

Themes	Highlights	Studies and regions (selective)
The geography of network growth	Surface transportation networks subject to geographical constraints;	Gastner and Newman [20] (EU) Barrat et al. [6] (NA)
	Simulation models to replicate the evolution of transportation networks	Taaffe et al. [52] (WW) Pred [41] (NA) RIMMER [43] (OC) Farahani et al. [18] (EU) Jaržemskiene [27](WW)
Correlations among growth factors	Driving or interacting forces behind the expansion of transportation networks, e.g., land use, vehile miles traveled, economic development, etc.	Antrop [3] (EU) McKinnon [39] (EU) Shi et al. [48] (AS) Cervero and Hansen [10] (NA)
Analyses of topological properties	View it as a complex network	Tsiotas [55] (EU) Ingvardson and Nielsen [26] (EU)

AS—Asia; EU—Europe; NA—North America; OC—Oceania; WW—worldwide

### Understanding the evolution of micro-mobility systems

Early probe of micro-mobility systems is majorly centered on bike-sharing systems. Pioneering bike-sharing schemes are largely station-based, and the newest variant that becomes ubiquitous is fully dockless. This can be viewed as different generations of bike-sharing programs, a three-stage transformation as defined by DeMaio [15] and Shaheen et al. [47]. More recent efforts were directed to the growth of a single system. As shown in Table 2, such research can be dichotomized into two categories: the modeling and analysis of evolving bike-sharing systems; and the adoption of growth simulation to understand the demand/supply equilibrium of a system [35, 54]. For instance, Hamon et al. [22] converted bike-sharing networks over a period into dynamic graphs and conducted spectral analysis on the graphs. The results provided a visualization of evolving bike-sharing activities. Similarly, representing networks as signals, Borgnat et al. [7] found that the shared bike system in Lyon was a nonstationary evolution over the long term. Compared with these studies of the first category, investigating the dynamics of bike-sharing demands is more prevalent. Such research aims to understand the systematic equilibrium between trip demands and the supply of shared bikes. This process is examined over a short time scale, to demonstrate if the initial distribution of shared facilities matches the imbalanced demands over space when the system evolves. For instance, Yoon et al. [62] developed a prediction model that described the evolution of a bike-sharing system within a short time window (1 h). Chiariotti et al. [13] used a birth-death approach to monitor bike stations’ docking capacities and constructed a network to determine the routes where bikes were redistributed among different stations. An essential aspect of these studies was to monitor bike stations’ docking capacities if rebalancing strategies were implemented.

**Table 2:** Global studies investigating the evolution of micro-mobility systems and networks

Themes	Highlights	Studies and regions (selective)
The evolution of micro-mobility systems and networks	Different generations of micro-mobility systems;	DeMaio [15] (WW) Shaheen et al. [47] (EU, NA, AS) Almannaa et al. [2] (NA)
	Modeling of a growing system	Hamon et al. [22] (EU) Gehrke and Welch [21](NA) He et al. [23] (EU) Wang and Lindsey [57] (NA)
Understand the supply and demand equilibrium from the lens of growth dynamics	Model trip demands at the station level	Yoon et al. [62] (EU) Yang et al. [61] (NA) [13] (NA)

AS—Asia; EU—Europe; NA—North America; OC—Oceania; WW—worldwide

In recent years many cities have made substantial progress towards mass adoption of e-scooters, power-assisted bikes (e-bikes), and other newer variants of micro-mobility sharing options. This mass adoption provides big data about these emerging transport modes, facilitating a proliferation of publications recently. Several directions have been investigated, including user behaviors [32], spatiotemporal patterns [9, 24], and environmental impacts of these new systems [46]. Research on the network evolution of e-scooter sharing is still limited, although a particular interest is to compare e-scooter/e-bike sharing with conventional bike-sharing. The former’s advantages lie in better performance regarding sharing frequency, fleet size [64], and better replacement potential of first and last-mile or short trips [8, 60], compared with bike-sharing.

In summary, previous efforts have been dedicated to the understanding of various aspects of micro-mobility sharing systems, including network evolution process, supply and demand equilibrium, and the comparison of different sharing systems. This work complements the literature and goes further to (1) explore the long-term periodicity of a micro-mobility system; and (2) delineate its growth mechanism, i.e., the network evolution process of connections among sharing stations or service points.

## Method

### The framework

This study develops a workflow to examine the evolution of a micro-mobility system. At a higher level, we investigate how daily trips vary periodically with a window of several years. Next, we simulate the cumulative connections of each station or service point over space and time, which is an indicator of the system’s evolution. A critical assumption is that every micro-mobility system can be somehow modelled as a station, or service points-based system. The network of a docked system can be represented easily, as the origins and destinations of trips are fixed. For dockless systems, a normal procedure is to impose grid cells onto a study area and treat the centroids of each cell as a station or service point. There are also other approaches to represent a dockless system through a voronoi diagram based on a bus stop network for example [50]. These generated centroids can be thought of as hypothetical stations for a dockless system.

**Fig. 1:**
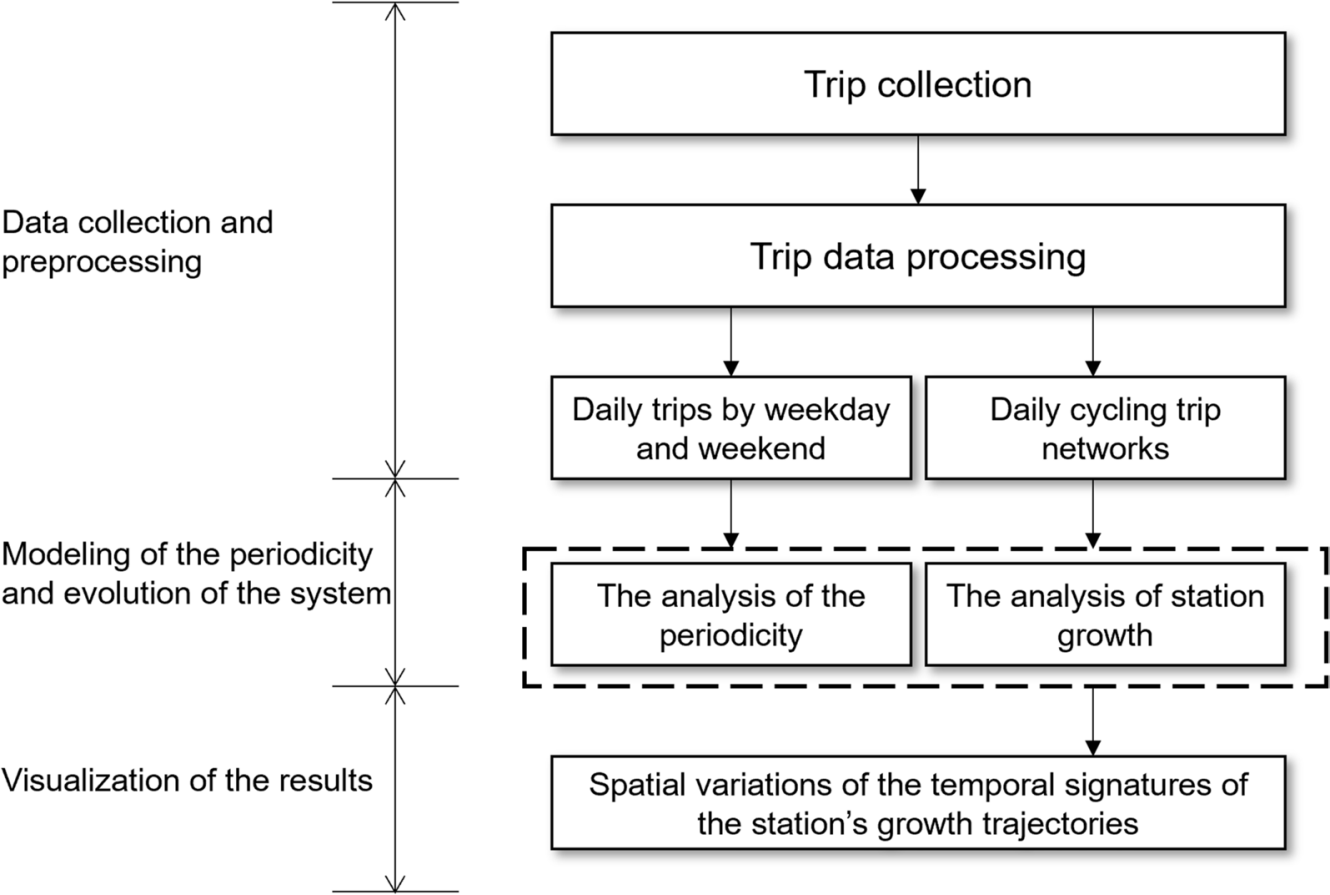
The logical framework of the proposed approach

The logical diagram of the proposed framework is displayed in Fig. 1. It contains three sequential components: data collection and preprocessing, the modeling of the periodicity and evolution of the system, and geo-visualization of the results.

*Data collection and preprocessing*. The data on cycling trips come with noise. Therefore, the first step is to eliminate abnormal trips. First, trips that were made from/to any “testing” stations were excluded from analysis. Second, trips with excessively short duration (e.g., less than 1 min) were also removed. These trips are largely due to false starts or users attempting to re-dock a bike. Finally, trips with average speeds exceeding local biking speed limit were removed as well, which were probably made by restocking trucks carrying bikes to different stations. After the preprocessing, the outputs are (1) the aggregated number of daily trips and (2) daily traffic networks.

*The periodicity and evolution of the system*. Two tasks are fulfilled. First, a Gaussian Mixture Model is applied to the data set of daily trips to identify yearly patterns of trip demands. Second, an eigendecomposition approach is used to analyze the hidden structures of each station’s growth trajectories, which is compared with the curve fitting results based on exponential and logistic models.

*Geo-visualization of the results*. The growth patterns of each station are spatially diversified. This is illustrated by the geo-visualization of top principal components (PC) extracted from the growth trajectory data. It can uncover the distinct growth landscape of different stations over the space.

### Traffic network

We can represent the system as a network where nodes are stations and edges denote the connections among the nodes. For a dockless system, hypothetical stations can be generated using approaches discussed in Sect. 3.1. Thus, a micro-mobility system can be denoted as a undirected network. A network on the date index *i* is defined as

1 $$\begin{aligned} BS_i = \{V,E\} \end{aligned}$$

where *V* is a set of the docking stations, and *E* denotes a set of edges. Specifically, the edges can be defined by

2 $$\begin{aligned} E = V \times V = \{e_{ij}\} \end{aligned}$$

where $e_{ij}$ is the link connecting station *i* and *j*. As we only consider the connections among different stations, traffic flows are ignored. Thus, $e_{ij}$ is 1 if there is at least 1 trip appearing on this edge, and 0 otherwise. In a network on a specific date, there is a set of connected nodes for each station. Therefore, for a certain time window, the set of the growth trajectories of station *i* can be described by

3 $$\begin{aligned} G_i = \{g_i | g_i, i = 1, 2, 3,\ldots ,n\} \end{aligned}$$

where $g_i$ is the number of cumulative connections on date index *i*, and *n* is the number of dates during the growth period.

### Prediction of the periodic of the system

**Fig. 2:**
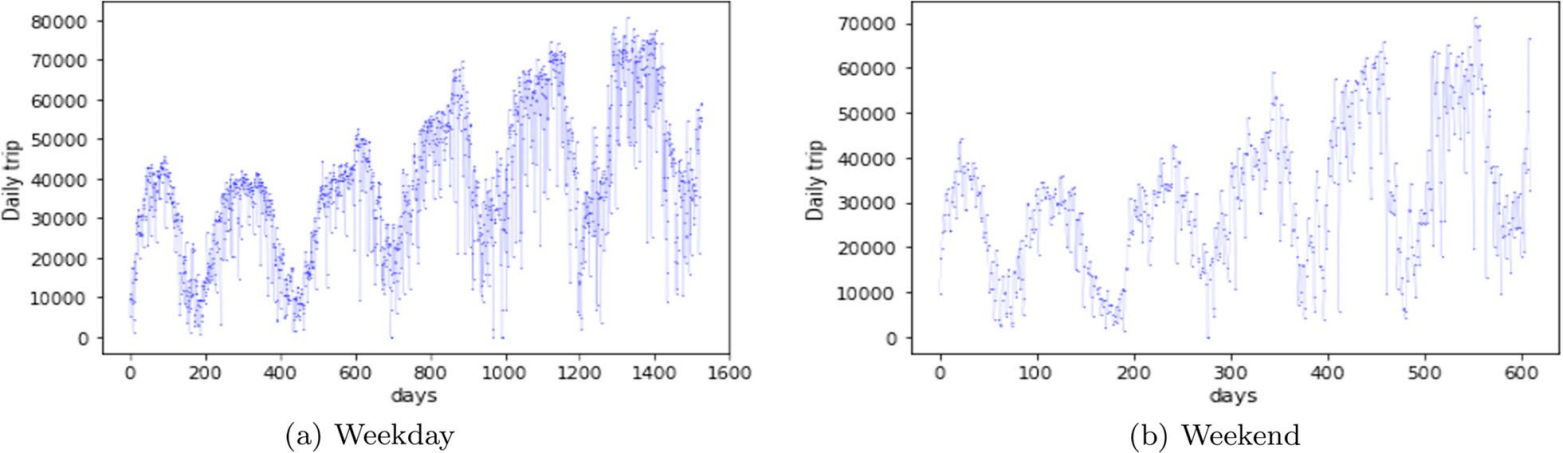
The temporal patterns of the variations of bike-share trips

The trips on weekdays and weekends show an upward increase and multiple local peaks (Fig. 2). The characteristic of multiple local peaks can be identified by a Mixture-of-Gaussian model.

A Gaussian Mixture Model is an approach to reveal the underlying degree of freedom of an unlabeled data set. Its primary application is to identify a clustering structure of the data. It fits a probability density function that has different components represented by a Gaussian distribution. Each distribution is parameterized with a mean and a covariance matrix, and thus the entire data set can be represented by the specified Gaussian parameters. Each component can be thought of as a cluster centered at a peak point with the highest probability.

The simplest application of the Gaussian Mixture Models is to identify the clusters of a one-dimensional data set. It is employed by this work as well. Before fitting the probabilistic model, we need to convert the temporal trip data into an appropriate format that can be interpreted by the model. Take the weekday trips for example (Fig. 2a). The original data set can be expressed by

4 $$\begin{aligned} TP = \{TP_i|TP_i, i=1,2,\ldots ,n\} \end{aligned}$$

where $TP_i$ is the total number of weekday trips in date index *i*, and *n* is the total number of weekdays in the data set.

To model the trip distributions, we use a set of dates as a data representation method. The representation is defined by

5 $$\begin{aligned} W = \{W_i|W_i, i=1,2,\ldots ,n\} \end{aligned}$$

where $W_i$ is a vector $\mathbf {W_i}$ for weekday index *i*, in ${\mathbb {R}}^{d_i}$. The dimension $d_i$ is the number of trips for weekday *i*, and the number of element in the vector is *i*. For example, if in the first weekday $W_1$ there are 5000 trips, $\mathbf {W_1}$ is in ${\mathbb {R}}^{5000}$ and represented by $(\underbrace{1,1,\ldots ,1}_\text {5000})$. All the vectors are subsequently concatenated into a row vector that equivalently denotes the weekday set *W* as

6 $$\begin{aligned} W = (\underbrace{1,\ldots , 1,}_{d_1} \ \underbrace{2,\ldots , 2,}_{d_2} \ ,\ldots , \ \underbrace{n,\ldots , n,}_{d_n}) \end{aligned}$$

where the dimension of *W* is the total number of trips. $W^T$, a column vector, can be viewed as a one-dimensional data set in which each data point corresponds to a date index. We assume that the data follow a Gaussian distribution, as may indicated by Fig. 2. Thus, given one-dimension data set with data points $x_1,\ldots ,x_n \in \mathbb {R}^1$, we can fit it with a Gaussian Mixture Model *M*, which is parameterized by a set as

7 $$\begin{aligned} M = \{(\pi _i, P_i)|\pi _i, i=1,2,\ldots ,k; P_i = N(\mu _i, \rho _i^2),i=1,2,\ldots ,k\} \end{aligned}$$

where *k* is the number of Gaussian components, $\pi _i$ is the weights of different components, and a component has mean $\mu _i$, and $\rho _i^2$.

The probability of a data point *i* is defined as

8 $$\begin{aligned} Pr_i = \sum _{j}^{m}Pr(i, G_j) = \sum _{j}^{m}\pi _iPr(i|G_j) \end{aligned}$$

where $Pr(i|G_j)$ is the probability of *i* under the Gaussian distribution of *j*.

Furthermore, the probability of the data set is defined as

9 $$\begin{aligned}{}&Pr(data\vert \pi _1P_1+\cdots +\pi _kP_k) \\ &\quad = \prod _{i=1}^{n}(\pi _1P_1(x_i)+\cdots +\pi _kP_k(x_i)) \\ &\quad = \prod _{i=1}^{n}\left( \sum _{j=1}^{k}\frac{\pi _j}{(2\pi \rho ^2_j)^{1/2}} \hbox{exp} \left( -\frac{(x_i-u_i)^2}{2\rho ^2_i}\right) \right) \end{aligned}$$

The goal is to find a model *M* (Eq. 7) that maximize the function (Eq. 9). However, there is no optimum solution for this problem. So the expectation–maximization algorithm is employed to identify a local optimum solution. Given a data set with *n* data points of one dimension, the algorithm first initiates the Gaussian components randomly by k-means (another clustering algorithm, please refer to Arthur and Vassilvitskii [4]) or other methods. Next, it repeats the following two steps until convergence. The first step assigns each point $x_i$ fractionally to the *k* components, so the weight of $x_i$ associated with a component $P_j$ is

10 $$\begin{aligned} w_{ij} = Pr(P_j|x_i) = \frac{\pi _jP_j(x_i)}{\sum _{m}^{k}\pi _mP_m(x_i)} \end{aligned}$$

The second step is to update the model’s parameters:

11 $$\begin{aligned} \pi _j&= \frac{1}{n}\sum _{i=1}^{n}w_{ij} \end{aligned}$$

12 $$\begin{aligned} \mu _j&= \frac{1}{n\pi _j}\sum _{i=1}^{n}w_{ij}x_i \end{aligned}$$

13 $$\begin{aligned} \rho ^2_j&= \frac{1}{n\pi _j}\sum _{i=1}^{n}w_{ij}(x_i-\mu _j)^2 \end{aligned}$$

The model is a unsupervised learning task, and thus we do not know the true number of the Gaussian components. Hence, the elbow method is applied to identify an optimal number of the components. Specially, given a candidate set of the number of components, Akaike information criterion (AIC) scores are generated. According to the elbow method, the number associated with the steepest decrease of the AIC is believed to a local optimal solution.

### The modeling of the initial growth of the system

Based on network theories, we can regard the evolution of a micro-mobility system as the expansion of connections of each station over time. Equation 3 tracks the growth statistics for each station, which serves as a basis to model the system’s evolution. The system is periodic in terms of trip demands, and it keeps the upgrade and expansion of shared facilities. Such processes are complicated, so it is hard to investigate the system’s evolution over a full life cycle. Therefore, this work adopts a simplified approach by only examining the system’s initiating period, i.e., six months after the system is implemented.

#### Growth curves of connected stations

Many natural phenomena, such as population increase and bacteria reproduction, can be modeled by exponential or logistic growth. We hypothesize that the growth of a bike station may follow similar patterns. Therefore, the growth of each station is fitted against an exponential growth model defined by

14 $$\begin{aligned} g_i = C - ae^{-bi} \end{aligned}$$

where $g_i$ is the number of connections on date *i*, *C*, *a*, and *b* are model parameters.

The growth is also fitted against a logistic growth model, which is defined by

15 $$\begin{aligned} g_i = \frac{C}{1 + ae^{-bi}} \end{aligned}$$

For each station, the model with a higher R square is selected.

#### Analysis of the spatio-temporal growth patterns by eigendecomposition

The hidden structures of the data may dominate the growth trajectories of stations. An eigendecomposition method can reveal such hidden patterns. Thus, it has been in widespread applications in human mobility studies. It is used to extract top PCs from a data set, and the top PCs can explain the inherent variance of the data. The resultant PC coefficients can represent the original data well. The eigendecomposition is a dimension reduction approach and particularly appropriate for those data sets with high dimensions. Additionally, the growth patterns of different stations can be reconstructed by the top few PCs and associated coefficients. The reconstruction may indicate how the patterns deviate from the average growth trajectory.

Specifically, each station’s growth can be represented by a vector $\mathbf {A_i} = \{a_{i1},a_{i2},\ldots ,a_{im}\}$ where $a_{ij}$ refers to the number of connections of station *i* on date *j*, and *m* is the number of dates. Thus, the average vector $\mathbf {\mu }$ can be obtained by

16 $$\begin{aligned} \mathbf {\mu } = \frac{1}{N}\sum _{i=1}^{N}\mathbf {A_i} \end{aligned}$$

where *N* is the total number of stations during the growth period. A station’s temporal deviation from the average vector is $\mathbf {A^{'}_i} = \mathbf {A_i} - \mathbf {\mu }$. A matrix *K* can be then defined by

17 $$\begin{aligned} K_{N \times M} = \begin{bmatrix} a^{'}_{11} &{}\quad a^{'}_{12} &{}\quad \dots &{}\quad a^{'}_{1m}\\ a^{'}_{21} &{}\quad \ddots &{}\quad &{}\quad a^{'}_{2m} \\ \vdots &{}\quad &{}\quad &{}\quad \vdots \\ a^{'}_{n1} &{}\quad a^{'}_{n2} &{}\quad \dots &{}\quad a^{'}_{nm} \\ \end{bmatrix} \end{aligned}$$

where *M* is the number of dates. A covariance matrix *C* of size $M \times M$ is an averaged outer product of *K*, which is computed by

18 $$\begin{aligned} C = \frac{1}{N}K^TK \end{aligned}$$

The covariance matrix is used to calculate all the eigenvectors (set $\{\mathbf {e_i}|\mathbf {e_i}, i=1,2,\ldots ,m\}$) and associated eigenvalues ranked by descending order (set $\{\lambda _i|\lambda _i, i=1,2,\ldots ,m\}$). The eigenvectors represent the PCs of matrix *K*. A coefficient matrix *B* can be computed by

19 $$\begin{aligned} B = KE^T \end{aligned}$$

where *E* is a matrix whose rows denote the eigenvectors, and the resultant matrix *B* is denoted as

20 $$\begin{aligned} B_{N \times M} = \begin{bmatrix} b_{11} &{}\quad b_{12} &{}\quad \dots &{}\quad b_{1m} \\ b_{21} &{}\quad \ddots &{}\quad &{}\quad b_{2m} \\ \vdots &{}\quad &{}\quad &{}\quad \vdots \\ b_{n1} &{}\quad b_{n2} &{}\quad \dots &{}\quad b_{nm} \\ \end{bmatrix} \end{aligned}$$

where $b_{ij}$ denotes the coefficient of the *j*th PC for station *i*. The top few coefficients are important. They are used to reconstruct the original temporal signatures of the growth of a station, which is computed by

21 $$\begin{aligned} \mathbf {A_i} = {\boldsymbol{\mu}} + B_iE \end{aligned}$$

Empirical rules are normally adopted to determine the number of the top PCs applied in the reconstruction process. For this study, we only retain the first few PCs that account for at least 90% of the total variance.

## Case study

### New York bike-sharing system and data sources

**Fig. 3:**
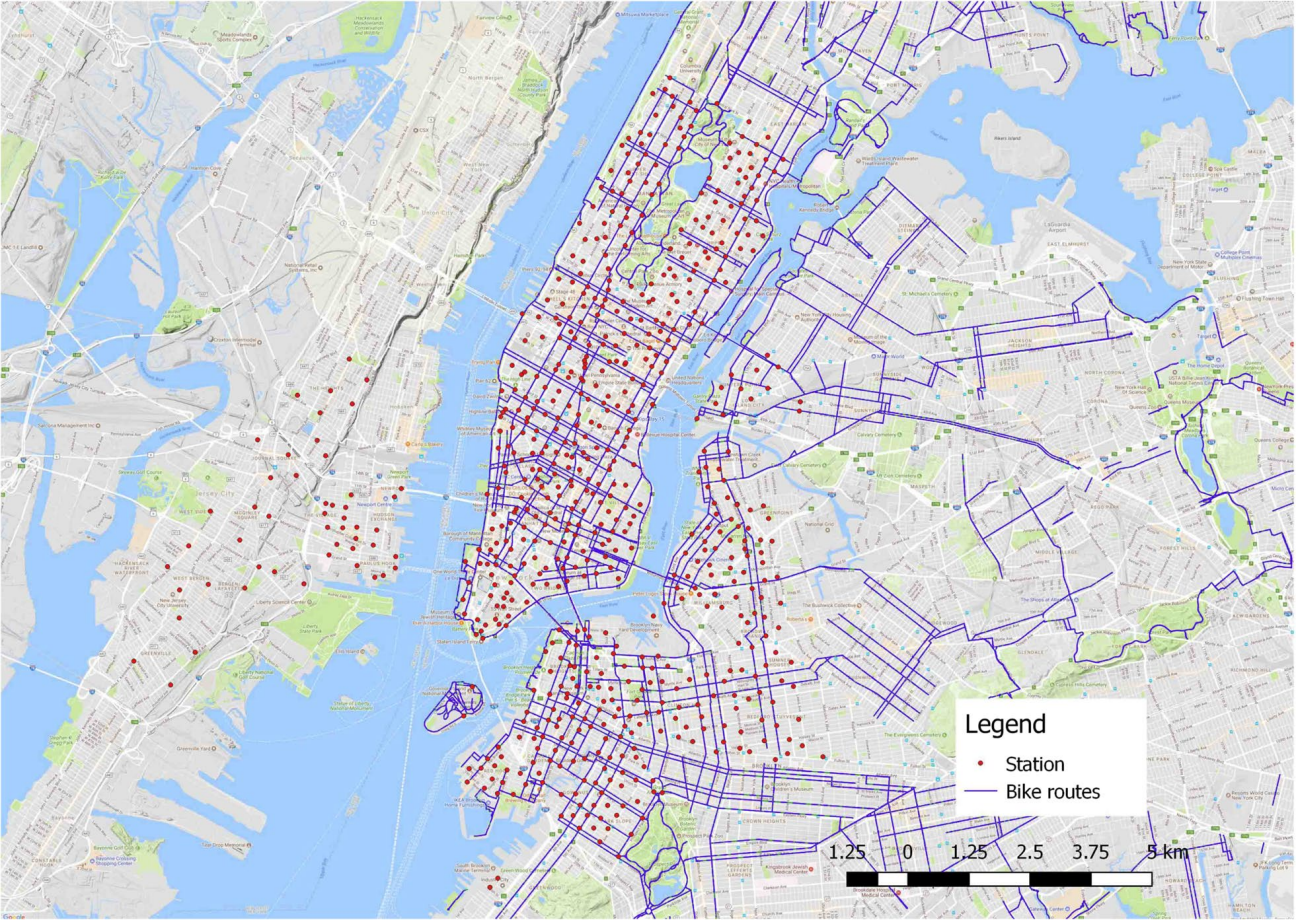
The study area

We applied the developed framework into Citi Bike, a bike-sharing program in New York City, USA. Citi Bike is a docked system and started to operate as early as May 2013 and has become one of the most successful bike-sharing schemes globally. Citi bike started with around 330 stations, and as for 2021 (the time of writing) it has nearly 950 stations and over 14,000 public bikes. Its service areas cover Manhattan, Brooklyn, Jersey City, and other urban districts in the city, as displayed in Fig. 3.

Data were collected from multiple open sources. Bike-sharing trip data were fetched from Citi Bike’s official website (https://www.citibikenyc.com/). The website maintains an online repository of trip information that is updated monthly. Each trip has 11 attributes: start/stop time and location, user type, gender, and other information. The information about metro lines and stations were downloaded from two open data sources: https://data.ny.gov/ and https://opendata.cityofnewyork.us/. The bike-sharing data were well-prepared by the provider, and therefore the minimum amount of effort was put to pre-process the data. During the first five workdays, the bike-sharing operator tested the system extensively. Thus, the corresponding trip data were excluded from the analysis.

### The evolving and periodic characteristics of the system

**Fig. 4:**
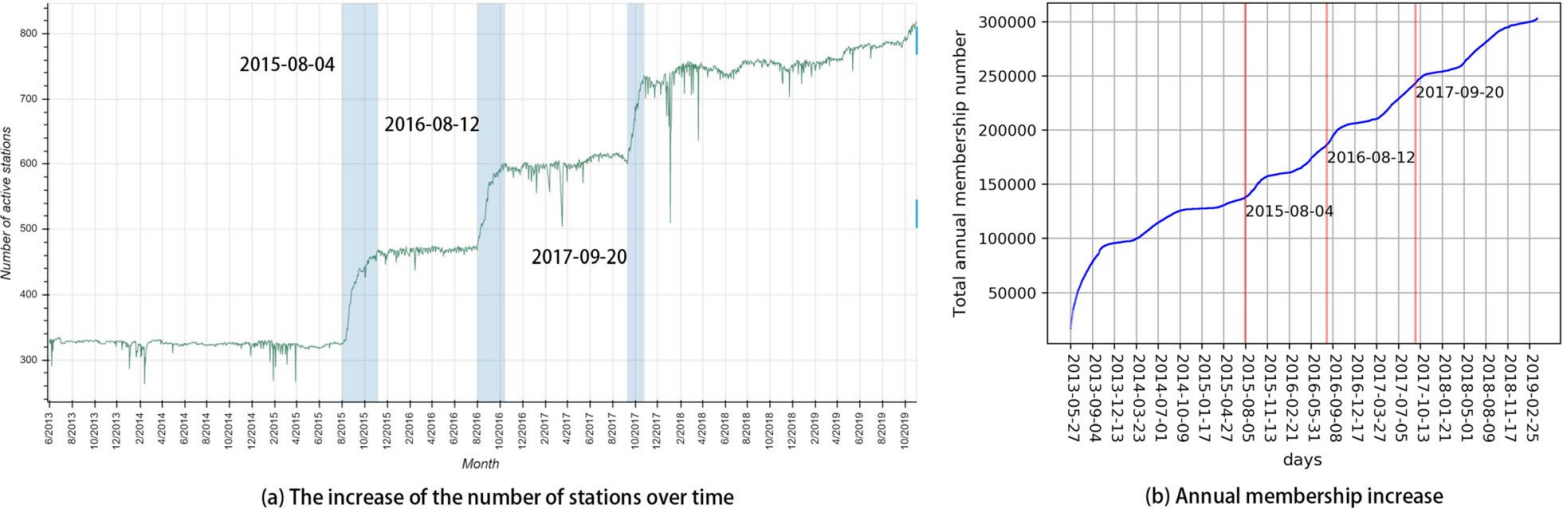
The increase of the number of stations and annual membership

**Fig. 5:**
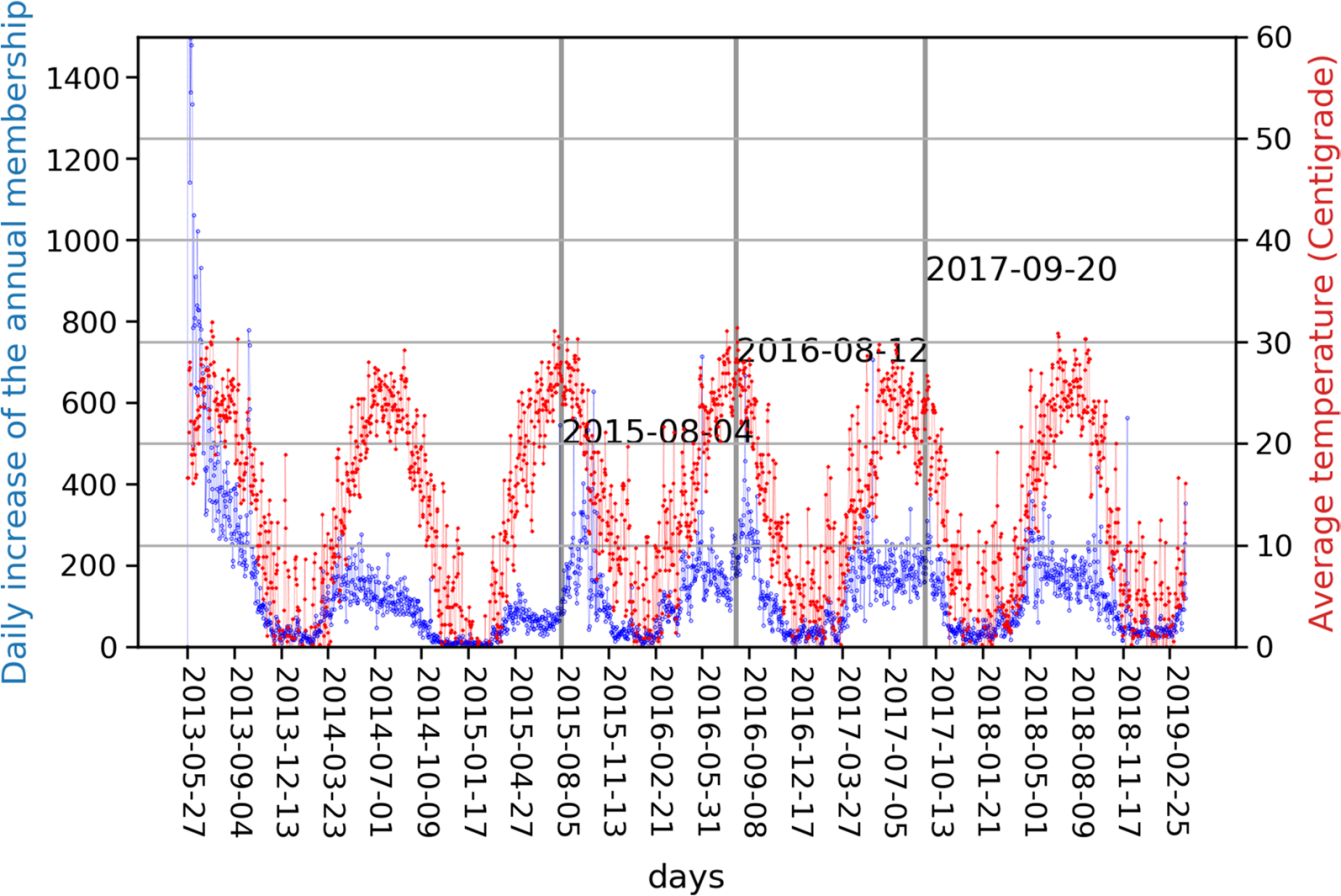
Temperature and daily annual membership increase

**Fig. 6:**
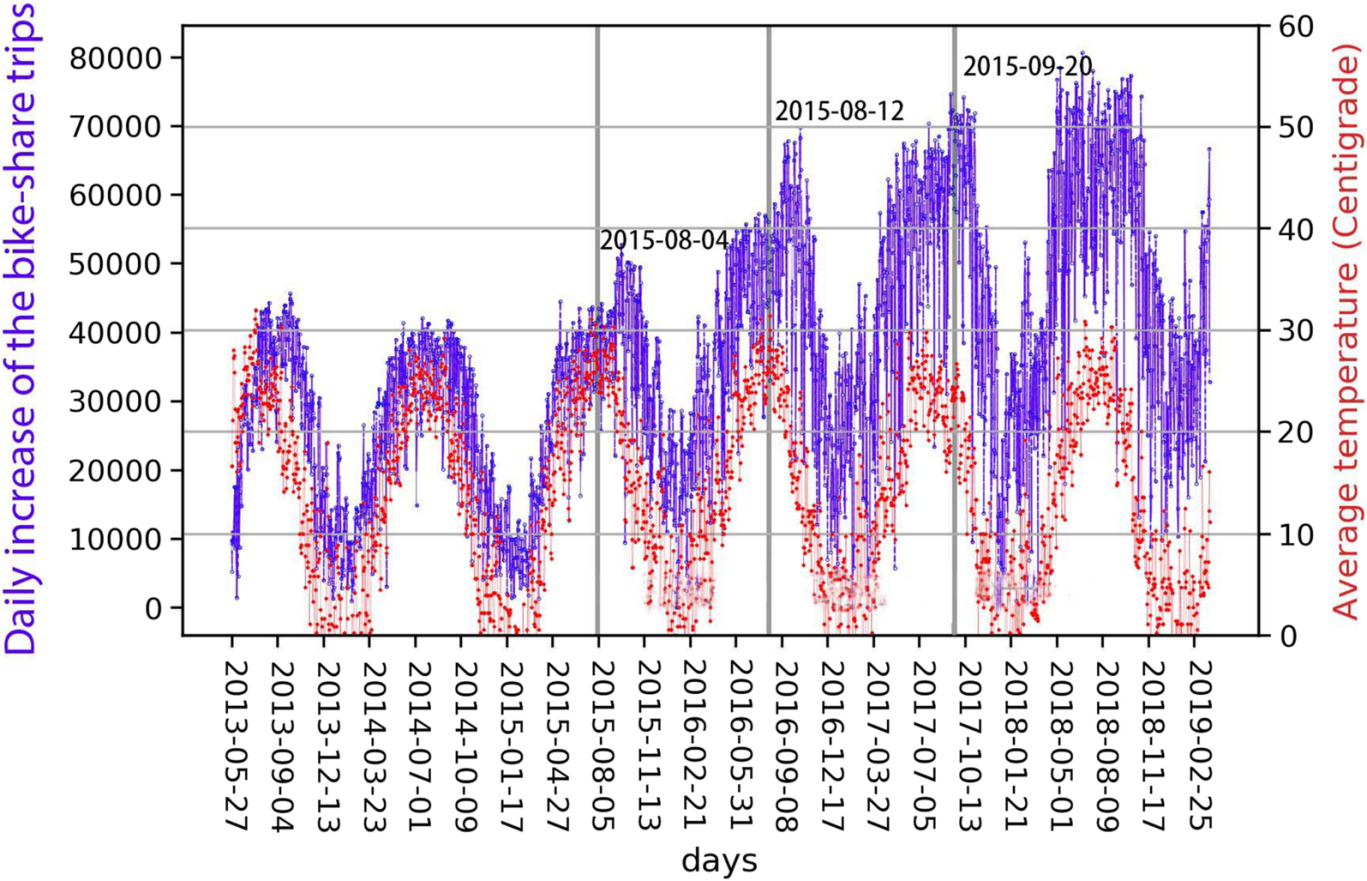
Temperature and daily trip

The bike-sharing system seems to be both expanding and periodic, which is a primary distinction from social networks illustrated by Kumar et al. [30] and Kossinets and Watts [29]. In other words, it may not be monotonically expanding, but rather it evolves with both expansion and contraction. It has an increased number of new bike stations and a higher level of annual subscribed users in the long run (Fig. 4). However, the increases exhibit fluctuated patterns, as shown by Figs. 5 and 6. There were three periods when a batch of new stations was deployed, as shown by the shaded boxes in Fig. 4a). Nevertheless, the temporal tendencies of the amount of daily membership and the number of daily trips are mainly periodic and follow temperature fluctuations. This may imply a periodic component in the long-term evolution of the bike-sharing system. The above observations point to a potential contribution of temperature to the system’s periodicity, which is yet proved due to the constrained scope of this study. However, While not demonstrated by this work, temperature, precipitation, wind, and other weather condition factors are suggested to have strong influence upon the seasonal fluctuations of bike-share usage [38, 45].

### Modeling the periodicity

**Fig. 7:**
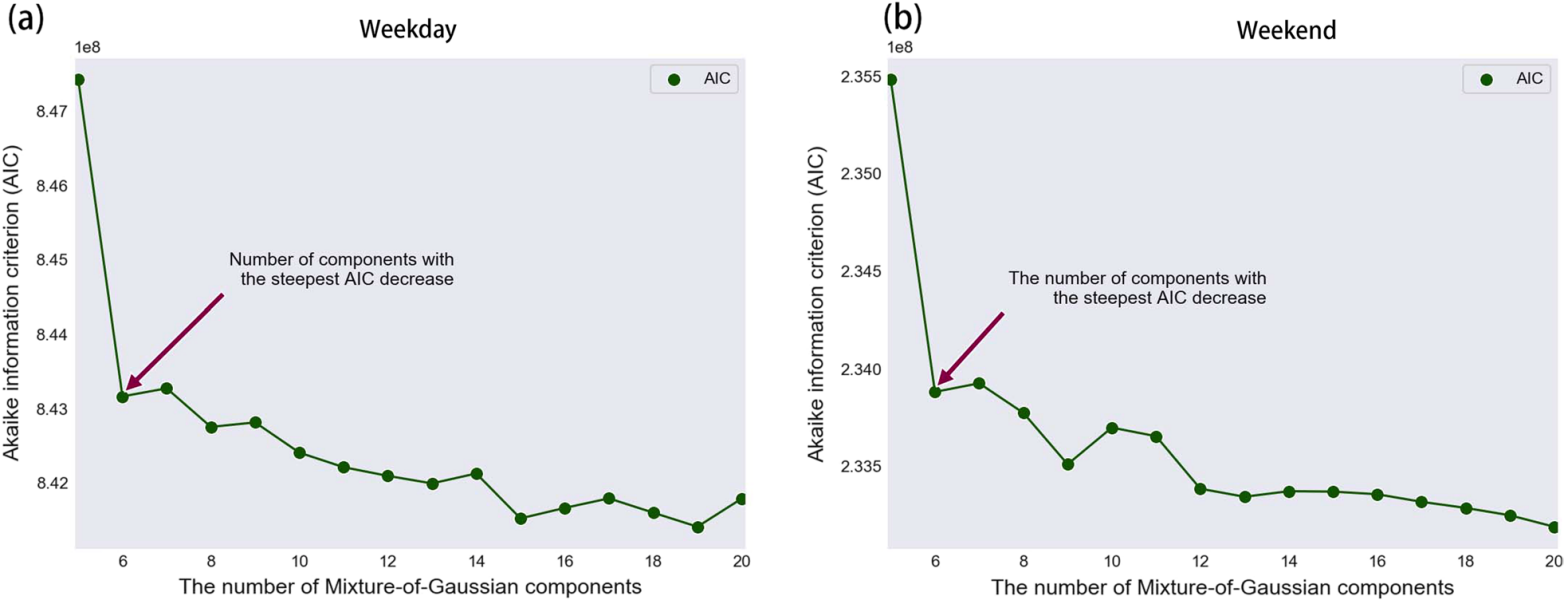
Akaike information criterion values for the weekday and weekend models. **a** Weekday. **b** Weekend

Figure 7 presents the AIC values associated with different numbers of Gaussian components. It indicates that six is the optimal number for both weekday and weekend cases. This number results in the steepest decrease in AIC.

**Fig. 8:**
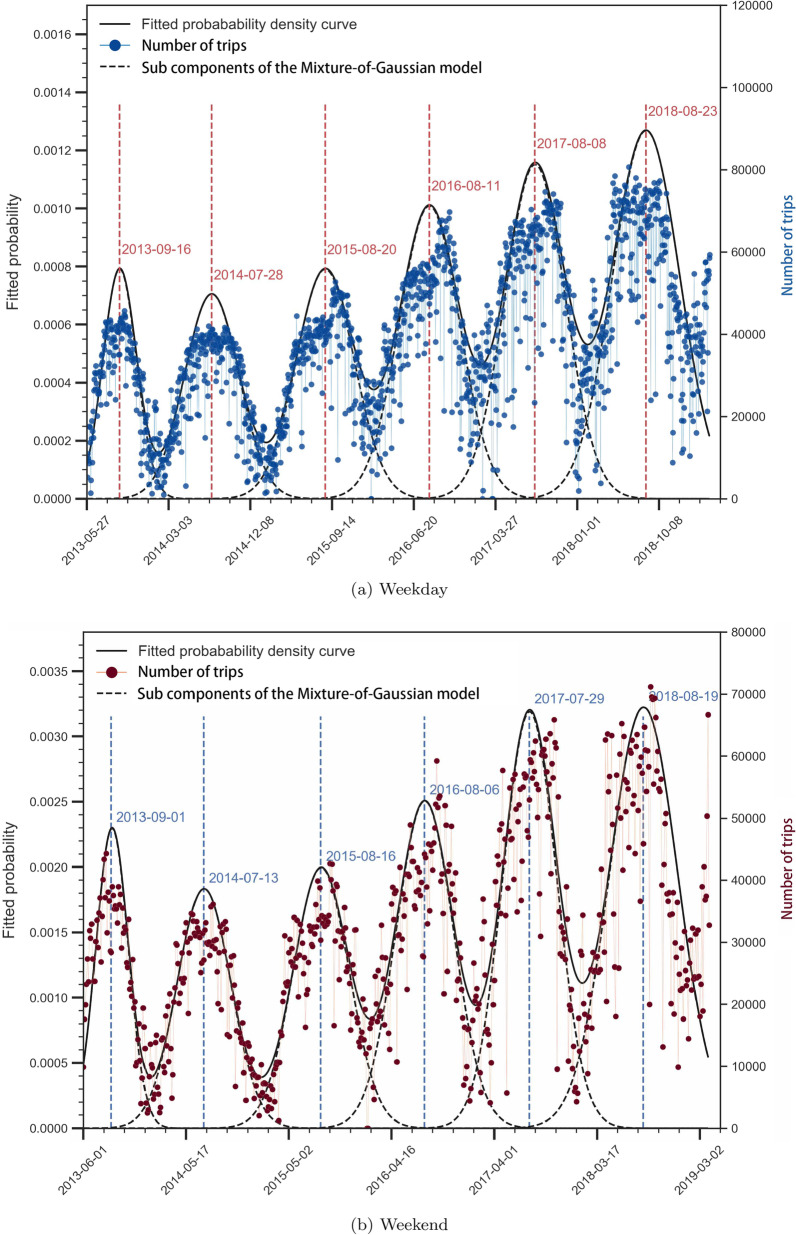
The growth periodic of New York’s bike-share system modeled by a Mixture-of-Gaussian model

The modeling results substantiate our observations in Sect. 4.2: (1) the periodicity is reflected by different Gaussian components; and (2) the trip demands climb in the long term (Table 3 and Fig. 8). The results also indicate that peak trip demands often occur in August or September. This is true for both weekday and weekend cases. While high demands on weekends appear within 20–30 days centered on the peak date, the range on weekdays is much wider, from 60 to 80 days. The modeled parameters can provide us with some hints of trip demands under future conditions.

**Table 3:** Parameters of the mixture-of-Gaussian model

Components	Weekday			Weekend		
	Peak	SD (in days)	No. of trips (on peak day)	Peak	SD (in days)	No. of trips (on peak day)
The 1st	2013-09-16	82	35,106	2013-09-01	26	28,330
The 2nd	2014-07-28	70	36,360	2014-07-13	26	27,089
The 3rd	2015-08-20	74	39,275	2015-08-16	27	30,337
The 4th	2016-08-10	41	50,100	2016-08-06	16	44,209
The 5th	2017-08-08	74	65,228	2017-07-29	33	52,955
The 6th	2018-08-23	66	73,877	2018-08-19	28	45,874

### The modeling and spatiotemporal patterns of initial growth of the system

**Fig. 9:**
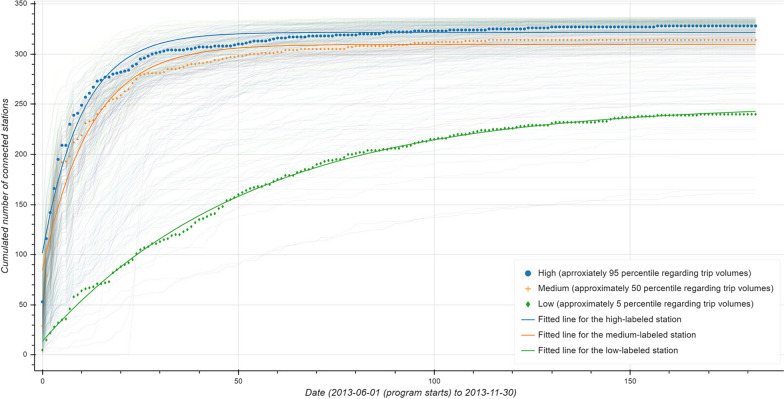
Raw growth trajectories and modeled curves of three representative stations

The growth of a station is modeled according to its connections with other stations over time. For example, for a station *A*, suppose on day 1 there were trips between this station and another station. On day 2, there were trips between station *A* and other two stations. The cumulative numbers were recorded, and therefore the growth trajectory of station *A* is from 1 to 3. The observations show that during the initiating period (from 2013-06-01 to 2013-11-30), the growth trajectory of all stations may follow two curves: exponential and logistic curves. While we verified such observations through modeling, over 90% of the stations follow the exponential growth. The majority of stations expand the connections with others very rapidly in the first couple of days, as shown by Fig. 9. The dotted lines refer to the growth trajectories of three representative stations with high (95 percentile), medium (50 percentile), and low (5 percentile) trip volumes, respectively. Those solid lines are fitted logistic curves. The models exhibit a reasonable fit.

The exponential and logistic growth models generate extremely high accuracy with an R square of more than 0.95. Table 4 gives three parameters of the models: limiting factor (C), growth factor (b), and initiating factor (a). The limiting factor controls the upper limit of the number of connections any station may have. The exponential model has a higher mean value of C (311.14) than the logistic. Secondly, the growth factors for both models are comparable. These modeling tools are powerful in capturing the initial expansion of a bike-sharing system. However, they only tell a part of the story because they fail to demonstrate the spatiotemporal signature of the growth trajectories. Thus, an Eigendecomposition analysis is introduced shortly to understand the evolution of the system better.

**Table 4:** The parameters of station growth models

Parameters	Exponential growth				Logistic growth			
	Mean	SD	Min	Max	Mean	SD	Min	Max
C (Limiting factor)	311.14	23.81	173.83	337.18	266.13	54.87	165.26	326.86
b (Growth factor)	0.08	0.03	0.01	0.21	0.16	0.08	0.04	0.34
a (Initiating factor)	242.16	34.00	144.66	323.94	672.43	2330.24	3.47	8426.48

**Fig. 10:**
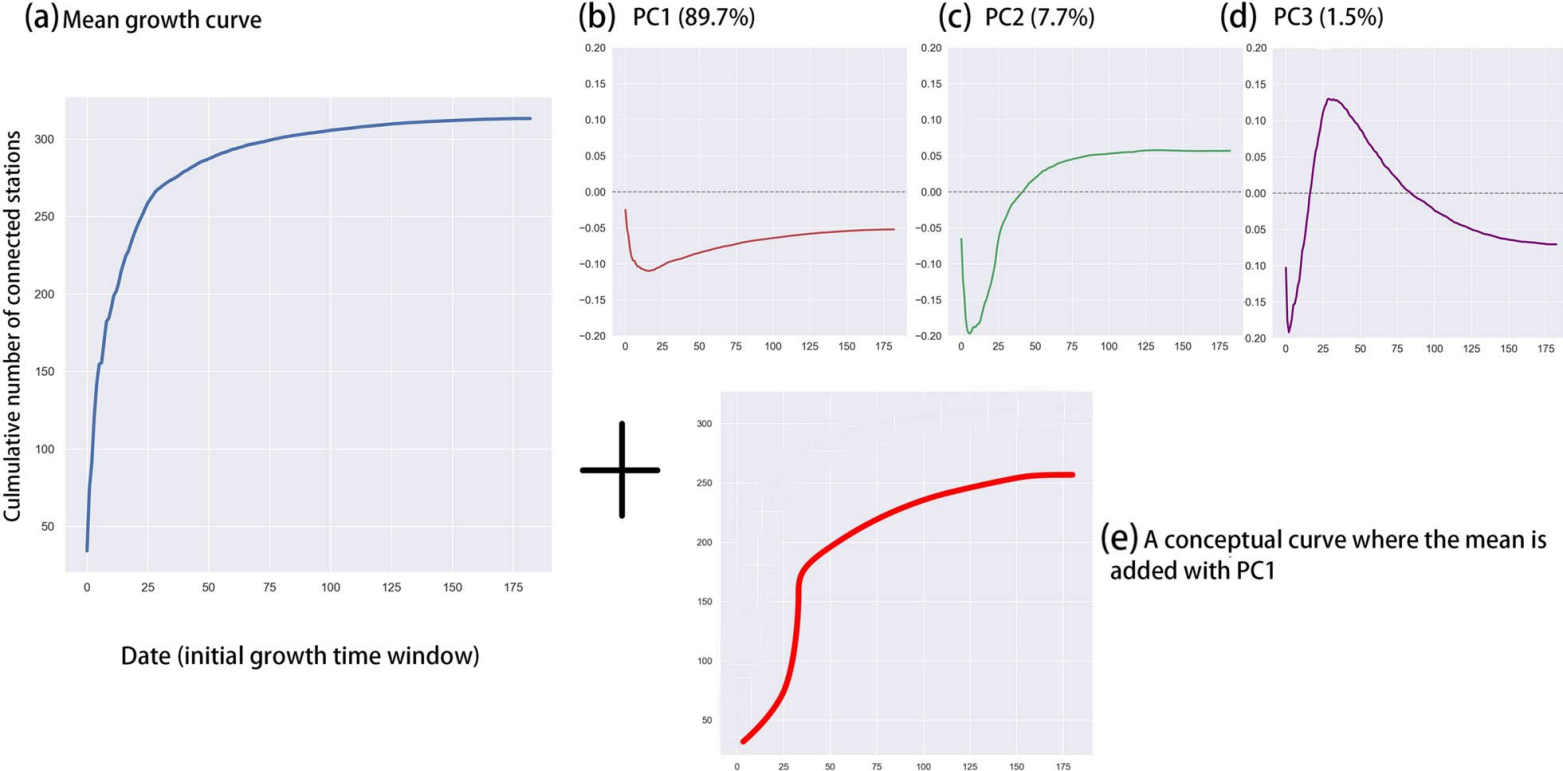
Eigendecomposition of station growth

The eigendecomposition of the temporal signatures of station growth can reveal crucial information about underlying structures. As for each station (i.e., observation), its data dimension is represented by 180 data indexes, constructed principal components (PC) can reveal interesting temporal growth patterns, in addition to their function as a dimension reduction tool. Figure 10 shows that the first three PCs explain over 95% of the total variance. These PCs also imply some temporal characteristics of station growth. First of all, Fig. 10a shows the base mode, i.e., a mean growth curve across all the stations. It is known that each PC adds an effect to the mean growth curve in order to reconstruct the original data. The first PC (Fig. 10b), explaining 89.7% of the total variance, represents a dominant effect that can be added onto the mean growth curve (Fig. 10a). According to 10b, PC1 looks very different with the mean growth curve. If PC1 is multiplied by a positive constant and added to the mean, the curve will be flattened out (Fig. 10e). In other words, if a coefficient of PC1 is large, growth will be greatly slowed down. Similar interpretation can be made to PC2 and PC3 as well. The 2nd PC (Fig. 10c) reveals another layer of the data structure. Some stations may gain an increased growth rate at the end of the growth cycle. This effect may partly explain why different growth curves are observed. While the 3rd PC may provide additional information, we do not elaborate because it only explains a negligible amount of the total variance.

**Fig. 11:**
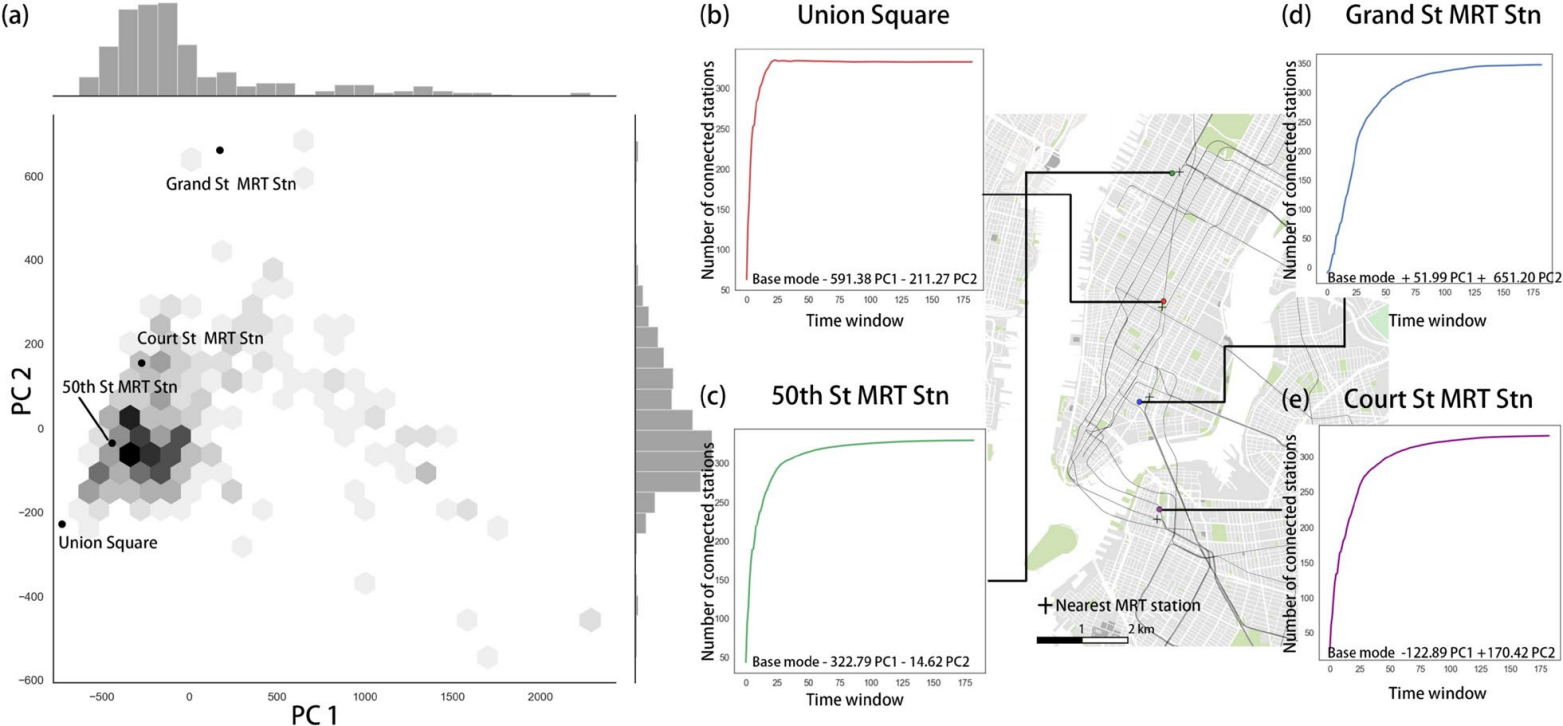
**a** Joint distribution of the coefficients of PC 1 and 2 of all stations and **b**–**e** temporal growth curves reconstructed from the two PCs

One strength of the eigendecomposition approach is that the original temporal signature of a station’s growth can be reconstructed by a linear combination of the base mode and the first few PCs weighted by accompanying coefficients. Figure 11a displays the joint distribution of the coefficients of the top two PCs for all stations. Four stations were selected to demonstrate how the reconstruction can be realized. Two stations (Fig. 11b,c) have higher-than-average trip demands, while the other two (Fig. 11d,e) have trip demands below the average. Union Square is situated in the heart of Manhattan, the CBD of New York City. It is surrounded by city parks, office buildings, and luxury condos. The 50th St MRT Station is located in northern Manhattan, next to Central Park, Time Square, and several residential buildings. Grand St MRT station is within the lower Manhattan. Court St MRT station is located in Brooklyn and close to several educational institutes.

High negative coefficients of both PC1 and 2 are associated with Union Square station. These negative values indicate a much higher growth rate during the first few weeks of operation. In other words, this station’s connections approach an upper limit within a short period, which can be seen from the recovered growth curve (Fig. 11b). The 50th St MRT station also has negative coefficients of both PC1 and 2. By contrast, the PC coefficients for the Grand St MRT station are positive, and the reconstructed curve seems to be an “S” shape (Fig. 11d). This shape suggests an inhibitive effect on the initial growth rate, which is reflected by the coefficient of PC 1. This station has a prolonged growth period, which should be related to the positive coefficient of PC 2. Lastly, Court St MRT station has both negative and positive PC coefficients. Note that the trip demand of this station is the lowest. This station is indeed a rare case, as shown by the joint distribution of the coefficients of PC1 and 2 (Fig. 10a).

**Fig. 12:**
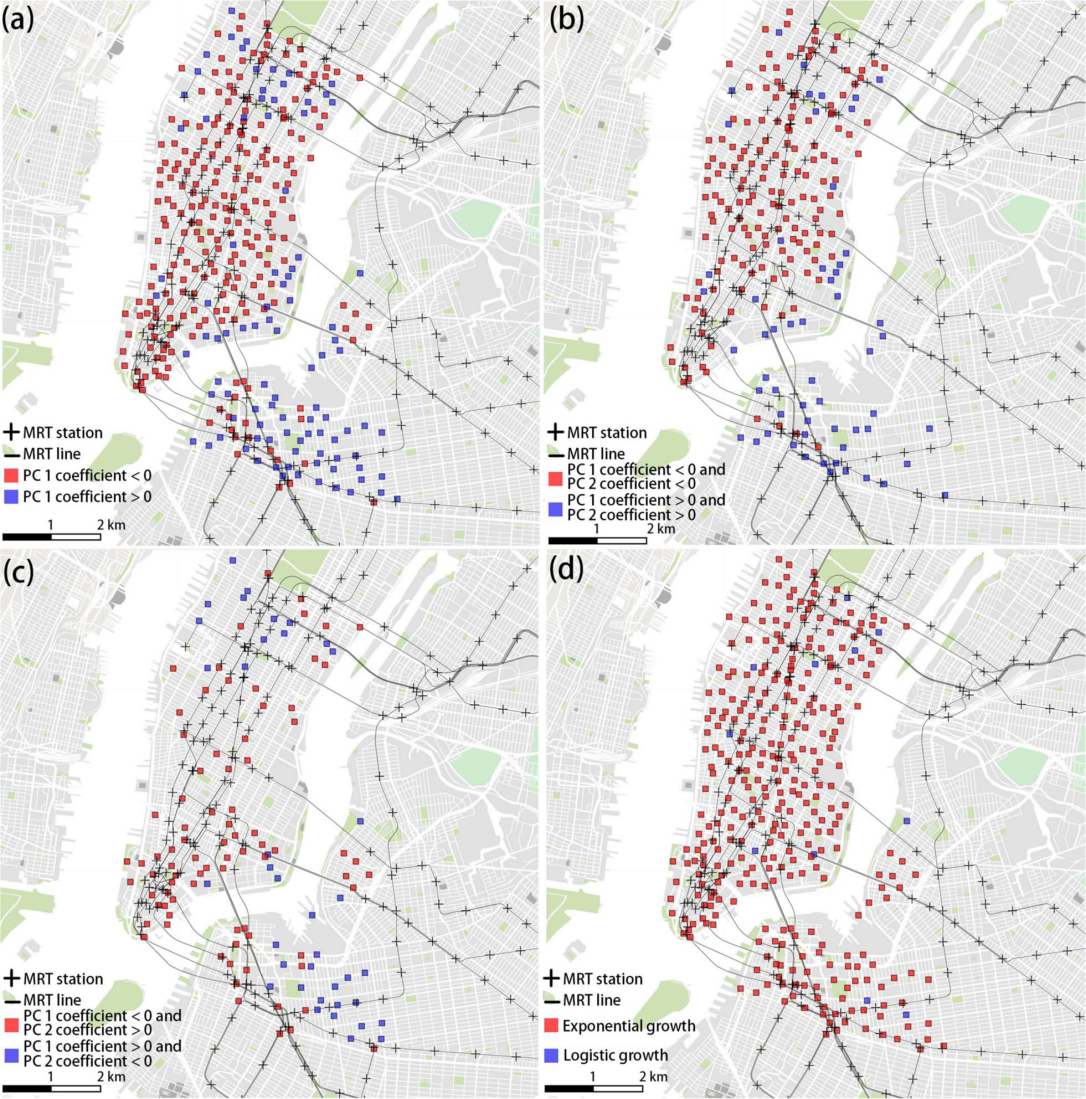
Distinct spatial patterns of station growth. **a** Negative and positive PC 1. **b**. Negative PC 1 and PC 2, Positve PC 1 and PC2. **c**. Negative PC 1 and positive PC 2; positive PC 1 and negative PC 2. **d** Exponential and logistic growth

From the joint distribution of the PC coefficients, we have demonstrated that the temporal signatures of growth curves of different stations are spatially diverse (Fig. 10). We further plotted four spatial maps that can provide a complete picture of the distinct growth patterns associated with different values of PC coefficients (Fig. 12). While the overwhelming amount of stations growth exponentially (Fig. 12d), a more subtle distinction of the growth patterns can be discerned based on the geovisualization of the eigendecomposition results (Fig. 12a–c). First, there is a dichotomized scenario of the growth rates of all stations when we categorize all the stations based on the coefficient signs of PC1 (Fig. 12). The stations with negative PC1 coefficients tend to have higher beginning growth rates than those with positive values. These stations are the majority and are located in Manhattan and immediate to multiple subway hubs. Considering both PC1 and PC2 coefficients, Fig. 12b displays that the stations with two positive coefficients are largely located outside the Manhattan district. These stations build connections and reach the maximum number of links relatively slower than those with both negative coefficients of both PC1 and 2. Figure 12c displays the distribution of stations with positive and negative signs of the two PC coefficients. It shows that the red squares accumulate around the left corner areas of Manhattan. These stations are featured by both strong initial and long-term growth potentials.

## Discussions and conclusions

The boom in the sharing economy and repaid advancement in the internet of things promote bike-sharing programs. This new transportation mode poses both challenges and opportunities for traffic operations and city management. Traditional transportation systems may be modified to incorporate the shared micro-mobility mode. To date, scientists have investigated micro-mobility systems in multiple directions, from systematic performance to usage patterns of cycling activities. Researchers also realize that these systems are not a stand-alone system and frequently interacts with built environment, public transit, and other urban components. The expansion and increased adoption of micro-mobility networks as a commuting mode motivates new research directions. One possible gap is the evolution of micro-mobility systems. Therefore, this work proposed a framework to uncover the periodic and evolving characteristics of a micro-mobility system and applied it into Citi Bike, a docked bike-sharing scheme in New York City, USA. A 7-year data set of cycling trips was collected to model the periodicity of the system through a Gaussian Mixture Model. Furthermore, a 6-month data set after the system initiated in May 2013 was extracted to investigate the temporal signatures of the stations’ growth. Exponential and logistic models were employed to fit growth curves, and principal component analysis was used to uncover the hidden structures of the growth trajectories.

We hope this work contributes to existing scholarship and practice across European and other North American regions in a few aspects. First, we develop an analytical framework to strengthen our understanding of the evolution of a micro-mobility system. How the interactions within a social network develop temporally has been well-documented. Surprisingly, simple models have been justified to explain these systems’ evolution elegantly. Motivated by such research, we incorporate both clustering and growth models into the framework. We find that it can sufficiently describe the growth and periodicity of the bike-sharing system in New York City. Because large metropolises such as Paris, London or New York share common geographical and demographic backgrounds, it is expected that the proposed framework may be applied to big European cities as well after a few assumptions are set properly and the level of biases is still acceptable. For instance, people may depend more on private car commuting and show moderate interests towards sharing modes in some Northern European cities, and the applications of the proposed framework would have certain constraints.

Second, more large and mid-sized cities in Europe may expansion existing or launch new systems to promote low-carbon, epidemic-resilient, and health beneficial travel modes. While shared bikes and e-scooters are well-advocated and plenty of programs have gained momentum in France, UK, and other European countries, there still exists a gap in numerous mid-sized towns between public transit supplies and travel demands. With the strike of COVID-19, many municipalities see a window of opportunities to promote micro-mobility sharing as a bridge of the gap as it is evidenced that commuters start to use shared bike more frequently in order to avoid over-crowded buses or trains during this post-COVID-19 era [5]. Accordingly, planners and transport policy makers need a tool to assess the utilization of existing systems and forecast how new stations may experience a surge of demand after a new system is launched, which is a practical aim of this work.

Particularly, the case study illustrated in this work point to a few emerging findings, demonstrating how a newly launched system may grow spatially and temporally. Bike-share demands follow a seasonal pattern. The bike-sharing system in New York City normally reaches its highest utilization rate in August and September, requiring a matched facility supply. However, during winter times, demands for the system fall to the bottom. This offers a clue for bike-sharing operators to ensure the system runs both sufficiently and economically. During high-demand periods, the supply of bikes should meet trip demands adequately. When the demand is low, operators should avoid the oversupply of facilities. The growth patterns of a bike-sharing system are critical for planning practices. For one thing, cities are planned so that residential areas, road networks, green space, and other sub-components expand to meet the developmental demand under future scenarios. Now it is the time to incorporate the new bike-sharing system into planning as it grows too. For another, cycling activities may occur among the stations with high growth potentials, which means that these stations are likely to build many connections rapidly. Thus, planning for cycling paths should also consider the growth of bike stations and potential linkages with other stations.

The case study also provides a multifaceted view of the system’s growth properties. It does not expand linearly. Instead, it exhibits a periodically increasing trend. The Gaussian Mixture Model identifies that the system is in high demand from around July to November each year, with the peak demand appearing in late August or September. Using the cumulative connections of each station as a quantity, we analyze the system’s evolution after its birth in 2013. The majority of the stations have growth trajectories that can be fitted using exponential models.

The growth trajectories are essentially the temporal signatures of the connections among stations. These temporal signatures may have hidden structures, which can be uncovered by the eigendecomposition method. Notably, two top PCs explain over 95% of the total variance. Specifically, the first PC has a dominant effect, explaining over 89% of the total variance. The temporal patterns of the 1st PC suggest that the change of growth rates during the early stage distinguishes the growth curves of different stations.

The coefficients associated with the top few PCs indicate how the temporal signatures of growth vary among different stations. Thus, we visualize the spatial distribution of stations with distinct temporal signatures. We find the stations with negative coefficients of the 1st PC dominates the Manhattan island, which means that these stations have rapidly increased growth rates shortly after the system is born. Moreover, the stations in the Bay area of Manhattan maintain a high level of growth rate during their entire life cycle. The distribution of the coefficients may be associated with built environment variables. For instance, Manhattan is the heart of New York City and contains office buildings, educational institutes, and multiple subway lines, high-density residential blocks. This level of land use mixture may contribute to the high growth potentials of the stations in this district. Additionally, the Bay area has the highest density of subway stations, potentially correlated with high-demand bike stations. However, more in-depth quantitative analysis is needed to verify such correlations.

The proposed framework may be used to evaluate the evolution of different micro-mobility systems as well. For example, do the stations in an e-scooter sharing system majorly follow an exponential growth curve? what about a hybrid system consisted of shared bikes, e-scooters, and e-bikes? It would be also beneficial to compare the network evolution processes between traditional bike-sharing and e-scooter sharing. E-scooter sharing exhibits different patterns regarding trip characteristics and user behaviors compared with bike-sharing, which may lead to different network evolution progresses. An important factor of the deployment of an e-scooter sharing system is overall energy consumption. A significant portion of energy loss results from idle status, and therefore e-scooter sharing systems always encourage a higher utilization frequency and a more compact fleet size than bike-sharing [58]. This may affect trip patterns and network evolution accordingly. Another outlook is about future micro-mobility system in particular and the whole urban transport system in general. This perspective may prompt a rethinking of established theories and heuristics regarding travel demand and commuter behaviors. An interesting line of inquires may be whether these new sharing options help to foster a more sustainable city via encouraging active mobility modes [17]. These new directions of investigations may be facilitated via the applications of the developed framework.

Admittedly, this framework has some and limitations. It is an analytical approach, rather than a simulation workflow. Bike-sharing systems are complex networks, and modeling its growth is sophisticated. Using the growth parameters verified in this study may be a start point to build a simulation model of system evolution. Secondly, we only tell the first part of the story and fail to explore a bike-sharing system’s full life cycle. We do not consider the following scenarios: the second batch of new stations is introduced to the system, or a new fleet of shared bikes is deployed. Finally, the application of Gaussian Mixture Models into simulating seasonal effects is debatable. While the clustering technique is model free, simple Trigonometric functions may be sufficient to model trip demands in different seasons. These are neglected points by the present work and yet a future research direction we aim to investigate.

## Data Availability

All data can be downloaded from https://www.citibikenyc.com/, https://data.ny.gov/, and https://opendata.cityofnewyork.us/.
